# Heart Rate and Use of Beta-Blockers in Stable Outpatients with Coronary Artery Disease

**DOI:** 10.1371/journal.pone.0036284

**Published:** 2012-05-03

**Authors:** Ph. Gabriel Steg, Roberto Ferrari, Ian Ford, Nicola Greenlaw, Jean-Claude Tardif, Michal Tendera, Hélène Abergel, Kim M. Fox, for the CLARIFY Investigators

**Affiliations:** 1 INSERM U698, Paris, France; 2 Université Paris Diderot, Paris, France; 3 AP-HP, Hôpital Bichat, Paris, France; 4 Department of Cardiology and LTTA Centre, University of Ferrara, Salvatore Maugeri Foundation, IRCCS, Lumezzan, Italy; 5 University of Glasgow, Glasgow, United Kingdom; 6 Montreal Heart Institute, Université de Montreal, Montreal, Canada; 7 Medical University of Silesia, Katowice, Poland; 8 NHLI Imperial College, ICMS, Royal Brompton Hospital, London, United Kingdom; Lerner Research Institute, Cleveland Clinic, United States of America

## Abstract

**Background:**

Heart rate (HR) is an emerging risk factor in coronary artery disease (CAD). However, there is little contemporary data regarding HR and the use of HR-lowering medications, particularly beta-blockers, among patients with stable CAD in routine clinical practice. The goal of the present analysis was to describe HR in such patients, overall and in relation to beta-blocker use, and to describe the determinants of HR.

**Methods and Findings:**

CLARIFY is an international, prospective, observational, longitudinal registry of outpatients with stable CAD, defined as prior myocardial infarction or revascularization procedure, evidence of coronary stenosis of >50%, or chest pain associated with proven myocardial ischemia. A total of 33,438 patients from 45 countries in Europe, the Americas, Africa, Middle East, and Asia/Pacific were enrolled between November 2009 and July 2010. Most of the 33,177 patients included in this analysis were men (77.5%). Mean (SD) age was 64.2 (10.5) years, HR by pulse was 68.3 (10.6) bpm, and by electrocardiogram was 67.2 (11.4) bpm. Overall, 44.0% had HR≥70 bpm. Beta-blockers were used in 75.1% of patients and another 14.4% had intolerance or contraindications to beta-blocker therapy. Among 24,910 patients on beta-blockers, 41.1% had HR≥70 bpm. HR≥70 bpm was independently associated with higher prevalence and severity of angina, more frequent evidence of myocardial ischemia, and lack of use of HR-lowering agents.

**Conclusions:**

Despite a high rate of use of beta-blockers, stable CAD patients often have resting HR≥70 bpm, which was associated with an overall worse health status, more frequent angina and ischemia. Further HR lowering is possible in many patients with CAD. Whether it will improve symptoms and outcomes is being tested.

**Trial Registration:**

Controlled-Trials.com ISRCTN43070564

## Introduction

Coronary artery disease (CAD) is the leading cause of death worldwide [1], [2], yet there is a paucity of data regarding the clinical characteristics and management of outpatients with stable CAD. Most of the available data are from patients admitted for acute coronary syndromes or treated with percutaneous coronary intervention (PCI). In addition, data often originate from Europe or North America. The prospeCtive observational LongitudinAl RegIstry oF patients with stable coronary arterY disease (CLARIFY) registry was initiated to improve our knowledge about patients with stable CAD from a broader geographic perspective [3]. The main objectives of the registry are to define contemporary stable CAD outpatients in terms of their demographic characteristics, clinical profiles, management, and outcomes; identify gaps between evidence-based recommendations and treatment; and investigate long-term prognostic determinants in this population.

Heart rate (HR) is a primary determinant of myocardial ischemia, and has been established as a prognostic factor in patients with CAD [4], [5], [6], [7], [8] and in those with congestive heart failure (CHF) [9]. It has also been correlated with the risk of future coronary events [4], [10]. Accordingly, the clinical benefits of beta-blockers in patients with CAD are well established, particularly the reduction in cardiovascular events in survivors of myocardial infarction [11].

Although beta-blockers have many actions other than simply reducing HR, emerging data show that HR reduction with pure bradycardic agents is also associated with clinical benefits, such as prevention of angina and reduction in myocardial ischemia [12], [13], [14]; and subset analyses from the BEAUTIFUL trial suggest that HR reduction may prevent coronary events [15], [16]. Despite these data indicating the prognostic impact of HR in CAD and the possible benefits of HR reduction, little is known regarding HRs actually achieved in clinical practice, including in patients receiving HR-reducing treatments such as beta-blockers. Likewise, there is a paucity of data on the management of elevated HR in patients with CAD in relation to the use of beta-blockers and other HR-reducing agents.

The goal of the present analysis is to describe, using a large contemporary database stemming from a broad geographic representation, the HR achieved in stable outpatients with CAD overall, and in relation to the use of beta-blockers, and to describe the determinants of HR. An additional goal is to assess the proportion of patients in whom resting HR exceeds some commonly described prognostic and therapeutic thresholds.

## Methods

### Study Design

CLARIFY is an ongoing international, prospective, observational, longitudinal cohort study in stable CAD outpatients, with 5 years of follow-up. The study rationale and methods have been published previously [3]. Patients were enrolled in 45 countries in Africa, Asia, Australia, Europe, the Middle East, and North, Central and South America. They are being treated according to usual clinical practice at each institution, with no specific tests or therapies defined in the study protocol.

### Study Population

Patients eligible for enrolment were outpatients with stable CAD proven by a history of at least one of the following: documented myocardial infarction (>3 months ago); coronary stenosis >50% on coronary angiography; chest pain with myocardial ischemia proven by stress electrocardiogram, stress echocardiography, or myocardial imaging; and history of coronary artery bypass graft surgery or percutaneous coronary intervention (performed >3 months ago).

Patients hospitalized for cardiovascular disease within the previous 3 months (including for revascularization), patients for whom revascularization was planned, and patients with conditions expected to hamper participation or 5-year follow-up (e.g. limited cooperation or legal capacity, serious non-cardiovascular disease, conditions limiting life expectancy, or severe cardiovascular disease [advanced heart failure, severe valve disease, history of valve repair/replacement, etc.]) were excluded from participating in the study.

### Site Selection

In order to enroll a population of stable CAD outpatients that mimicked the epidemiological patterns in each country, recruitment was based on a predefined selection of physician types (cardiologists, internists, primary care physicians) and aimed for consecutive enrollment of eligible patients. Physician selection was based on the best available sources, either local or regional, concerning the epidemiology and medical care data, including available market data and epidemiological surveys. A general target of 25 patients per million inhabitants was used (range 12.5–50) to ensure balanced representation of participating countries. Each physician recruited 10–15 outpatients with stable CAD, as defined by the inclusion criteria, over a brief period of time, in order to avoid selection bias.

### Baseline Evaluations and Data Management

Information collected at baseline included: demographics; medical history; risk factors and lifestyle; results of physical examination; HR (determined by both pulse palpation and the results of the most recent electrocardiogram [ECG] performed within the previous 6 months); current symptoms; laboratory values (e.g. fasting blood glucose, hemoglobin A1c [HbA1c], cholesterol, triglycerides, serum creatinine, and hemoglobin, if available); and current chronic medical treatments (i.e. those taken regularly by the patient, for ≥7 days before entry in the registry).

Data were collected centrally using an electronic, standardized, international case report form (translated into local languages) and sent electronically to the data management center where checks for completeness, internal consistency, and accuracy were run.

Data quality control is performed onsite in 5% of sites chosen at random in each country with, at each site, monitoring of 100% of case report forms for source documentation and accuracy. The study is being performed in accordance with the principles of the Declaration of Helsinki and was approved by the National Research Ethics Service, Isle of Wight, Portsmouth and Southeast Hampshire Research Ethics Committee, UK. Approval was also obtained in all 45 participating countries, in accordance with local regulations before recruitment of the first participant. All patients gave written informed consent to participate, in accordance with national and local guidelines. The CLARIFY Registry is registered in the ISRCTN registry of clinical trials with the number ISRCTN43070564.

### Statistical Analysis

All CLARIFY data are collected and analyzed at an independent academic statistics center at the Robertson Centre for Biostatistics, University of Glasgow, UK, which is responsible for managing the database, performing all analyses, and storing the data according to regulations. Baseline variables are summarized as means, standard deviations (SDs), medians, interquartile ranges (IQRs), and ranges for continuous data; and as counts and percentages for categorical data.

A multivariable analysis of independent correlates of HR≥70 beats per minute (bpm) was performed using a logistic regression model. The cutoff of 70 bpm was selected based on the results of several studies showing that it is an important prognostic threshold across a variety of patient populations [17], [18], [19], [20], [21]. All clinical baseline variables were considered for entry into the model as predictors of HR≥70 bpm and univariate models for each were produced. The use of HR-lowering medications was considered to be the most important treatment variable, and so this was the only treatment predictor entered in the analyses. The multivariable model was then built using a stepwise selection method applied to the remaining significant univariate predictors, with the use of HR-lowering medications being forced into the model.

## Results

**Figure 1:**
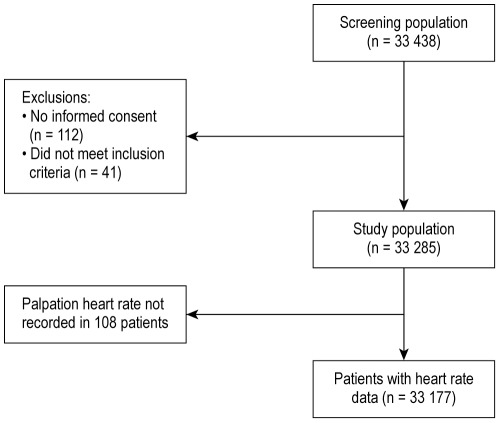
Patient flow chart.

**Figure 2:**
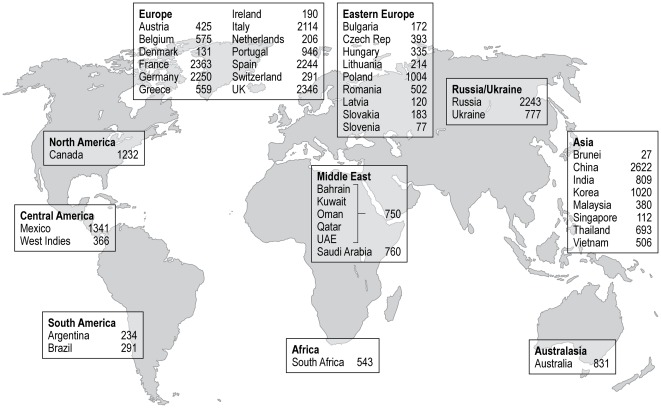
Geographical distribution of patients with baseline HR on palpation (*n* = 33,177).

A total of 33,438 patients were enrolled by 2,898 investigators in 45 countries between November 2009 and July 2010. Of these, 41 patients did not meet the inclusion criteria and 112 did not provide consent. Baseline data were available for 33,285 patients, of whom 33,177 patients had HR data recorded. Patient flow is depicted in Figure 1; and the geographic distribution of the study population is depicted in Figure 2.

**Figure 3:**
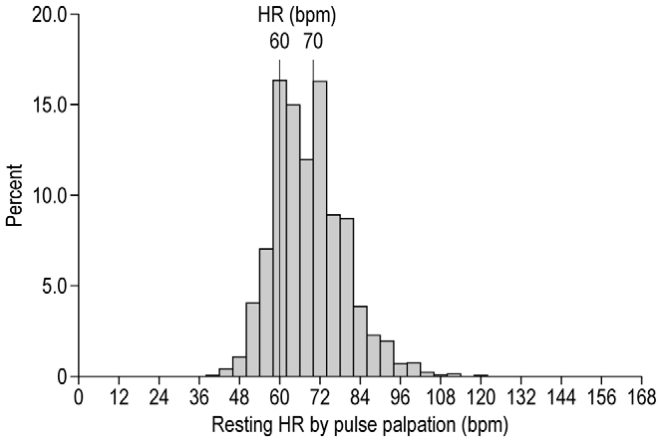
HR distribution in stable CAD patients.

The mean (SD) age was 64.2 (10.5) years and 77.5% of the patients were men (Table S1). The median time since the diagnosis of CAD was 5 years (IQR 2–9 years). Overall, 59.7% of the patients had a history of prior myocardial infarction and 58.7% had a history of PCI, while 23.4% had a history of coronary artery bypass grafting (CABG). A total of 22.0% of the patients had anginal symptoms; coronary angiography had been performed in 85.4% of the patients; and 61.9% of the patients had undergone a non-invasive test for ischemia. The mean (SD) pulse HR was 68.3 (10.6) bpm, while the ECG-derived HR was 67.2 (11.4) bpm. HR measured by pulse palpation was highly correlated to HR measured by ECG (correlation 0.81; *p*<0.0001). The distribution of HR as measured by pulse palpation is depicted in Figure 3.

Patients were divided in three mutually exclusive categories of baseline pulse palpation HR: ≤60 bpm (9,246, 27.9%), 61–69 bpm (9,322, 28.1%), and ≥70 bpm (14,609, 44.0%). The clinical characteristics of these three HR groups are described in Table S1. There were important, significant differences between the HR subgroups: patients with highest HR were younger, less frequently male or of Western descent, had less frequently undergone PCI or CABG, but had a more frequent history of hospitalization for CHF, stroke, history of asthma/chronic obstructive pulmonary disease (COPD), treated hypertension, diabetes, current smoking, and sedentarity (Table S1). They also had more frequent and more severe anginal symptoms and CHF symptoms (even though patients with Class IV New York Heart Association [NYHA] were excluded), and more frequent evidence of myocardial ischemia (Table S1).

The use of medications in the overall population and the three HR groupings is described in Table 1. With respect to HR-lowering medications, 75.1% of the population was treated with beta-blockers (any molecule and any dose), 9.8% received ivabradine, 2.5% digoxin or derivatives, 5.8% verapamil or diltiazem, and 2.9% amiodarone or dronedarone. The median number of HR-lowering agents used was one in the entire population and in each of the HR subgroups. Patients with higher HR less frequently received beta-blockers, but more frequently received ivabradine and digoxin, than patients with lower HR (Table 1). HR was higher as the number of HR-lowering medications increased above 1, from 67.6 (SD 10.3) bpm for patients receiving one agent, to 70.3 (11.8) bpm and 72.5 (12) bpm for patients receiving 2 and 3 HR-lowering agents, respectively (*p*<0.0001). The most commonly used beta-blockers were bisoprolol (34.1% of the patients), metoprolol tartrate (15.5%), atenolol (15.1%), metoprolol succinate (12.5%), carvedilol (11.6%), and nebivolol (5.6%). All other molecules were used in <2% of the patients receiving beta-blockers.

**Table 1:** Medications of the study population classified according to resting HR by palpation.

			Population According to Palpation HR			
Variable	Patients with Data	Total Population (*n* = 33,177)	≤60 bpm (*n* = 9,246)	61–69 bpm (*n* = 9,322)	≥70 bpm (*n* = 14,609)	*p*-Value
Aspirin, *n* (%)	33,157	29,068 (87.7)	8,071 (87.3)	8,257 (88.6)	12,740 (87.3)	0.0037
Thienopyridine, *n* (%)	33,111	8,959 (27.1)	2,561 (27.8)	2,493 (26.8)	3,905 (26.8)	0.21
Other antiplatelets, *n* (%)	33,108	3,069 (9.3)	742 (8.0)	875 (9.4)	1,452 (10.0)	<0.0001
Oral anticoagulants, *n* (%)	33,134	2,738 (8.3)	673 (7.3)	685 (7.4)	1,380 (9.5)	<0.0001
Beta-blockers, *n* (%)	33,161	24,910 (75.1)	7,390 (80.0)	7,281 (78.1)	10,239 (70.1)	<0.0001
Symptoms indicative of intolerance or contraindication to beta-blockers, *n* (%)	33,149	4,783 (14.4)	1,451 (15.7)	1,202 (12.9)	2,130 (14.6)	<0.0001
Ivabradine, *n* (%)	33,160	3,259 (9.8)	677 (7.3)	757 (8.1)	1,825 (12.5)	<0.0001
Calcium antagonists, *n* (%)	33,155	9,038 (27.3)	2,344 (25.4)	2,525 (27.1)	4,169 (28.6)	<0.0001
Verapamil or diltiazem, *n* (%)	33,155	1,931 (5.8)	421 (4.6)	491 (5.3)	1,019 (7.0)	<0.0001
ACE inhibitors, *n* (%)	33,160	17,044 (51.4)	4,792 (51.8)	4,835 (51.9)	7,417 (50.8)	0.15
Angiotensin II receptor blockers, *n* (%)	33,156	8,800 (26.5)	2,354 (25.5)	2,430 (26.1)	4,016 (27.5)	0.0012
Lipid-lowering drugs, *n* (%)	33,163	30,606 (92.3)	8,710 (94.2)	8,634 (92.7)	13,262 (90.8)	<0.0001
Long-acting nitrates, *n* (%)	33,156	7,329 (22.1)	1,761 (19.1)	1,999 (21.5)	3,569 (24.5)	<0.0001
Other antianginal agents, *n* (%)	33,151	4,618 (13.9)	999 (10.8)	1,284 (13.8)	2,335 (16.0)	<0.0001
Diuretics, *n* (%)	33,156	9,695 (29.2)	2,478 (26.8)	2,600 (27.9)	4,617 (31.6)	<0.0001
Other antihypertensive agents, *n* (%)	33,156	2,277 (6.9)	602 (6.5)	604 (6.5)	1,071 (7.3)	0.011
Digoxin and derivatives, *n* (%)	33,158	837 (2.5)	149 (1.6)	169 (1.8)	519 (3.6)	<0.0001
Amiodarone/dronedarone, *n* (%)	33,151	966 (2.9)	344 (3.7)	234 (2.5)	388 (2.7)	<0.0001
Other antiarrhythmics, *n* (%)	33,151	305 (0.9)	99 (1.1)	78 (0.8)	128 (0.9)	0.20
Antidiabetic agents, *n* (%)	33,160	8,153 (24.6)	1,698 (18.4)	2,186 (23.5)	4,269 (29.2)	<0.0001
Thyroid HRT, *n* (%)	33,157	1,422 (4.3)	427 (4.6)	393 (4.2)	602 (4.1)	0.17
Number of antianginals, median (IQR)	33,177	1 (1–2)	1 (1–2)	1 (1–2)	1 (1–2)	<0.0001
Number of HR-lowering agents, median (IQR)	33,177	1 (1–1)	1 (1–1)	1 (1–1)	1 (1–1)	0.0119
Number of antianginals or HR-lowering agents, median (IQR)	33,177	1 (1–2)	1 (1–2)	1 (1–2)	1 (1–2)	<0.0001

ACE, angiotensin-converting enzyme; HR, heart rate; HRT, hormone replacement therapy; IQR, interquartile range.

**Figure 4:**
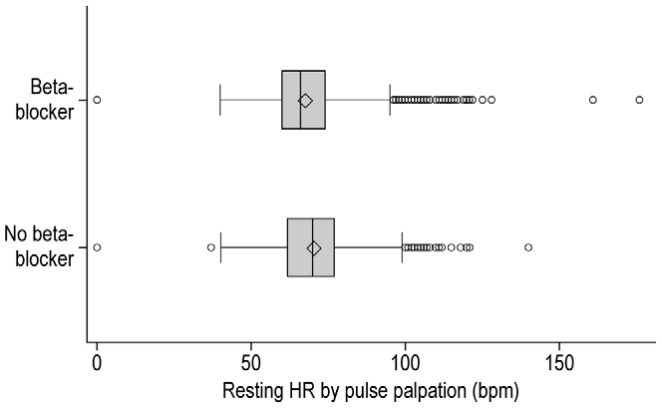
Distribution of HR for patients with versus without beta-blocker use. The vertical lines represent the minimum and maximum values. The box represents the lower (25^th^ percentile) and upper (75^th^ percentile) quartiles. Within the box, the vertical line is the median and the diamond the mean. Values>1.5 times the interquartile range were considered outliers and are shown as individual circles.

The patient population was also divided according to the use of beta-blockers. Baseline characteristics of patients receiving and not receiving any dose of beta-blockers are depicted in Table S2. Overall, 75.1% of the population received beta-blockers, but this proportion varied largely with co-morbidities: e.g. among patients with asthma, 50.9% were on beta-blockers. Patients receiving beta-blockers were significantly younger, more frequently diabetic, hypertensive, or dyslipidemic, and had a more frequent history of myocardial infarction, PCI or CABG, hospitalization for CHF, less frequent asthma/COPD, and more frequent anginal and CHF symptoms (Table S2). Systolic blood pressure was similar among patients with and without beta-blockers. The distribution of HR in patients treated or not with beta-blockers is depicted in Figure 4. Mean (SD) pulse HR was 67.6 (10.4) and 70.3 (11.2) bpm for patients with and without beta-blockers, respectively. The proportion of patients with HR≥70 bpm was 41.1% and 52.9% for patients with and without beta-blockers, respectively (*p*<0.001). Among 7,301 patients with anginal symptoms, 1611 patients (22.1%) had HR≤60 bpm.

A multivariable analysis was performed to identify the independent correlates of HR (Table 2). Among the most important predictors of elevated HR≥70 bpm were Asian ethnicity, asthma/COPD, diabetes, lack of use of HR-lowering drugs, increased diastolic blood pressure, angina class, hospitalization for CHF, and evidence of myocardial ischemia. Conversely, increasing physical activity was associated with decreased risk of elevated HR≥70 bpm.

**Table 2:** Factors associated with HR≥70 bpm.

Variable	OR (95% CI)	Pr>Chi-Square
Female sex	1.21 (1.14–1.28)	<0.0001
Age (per 10-year increase)	0.89 (0.87–0.92)	<0.0001
Ethnicity		<0.0001
Japanese/Korean	1.63 (1.41–1.88)	
South Asian	1.92 (1.76–2.11)	
Chinese	1.51 (1.38–1.67)	
Hispanic	1.43 (1.28–1.59)	
Black/African	0.88 (0.71–1.10)	
Unknown	0.80 (0.74–0.87)	
Angina CCS Class		0.018
I	1.07 (0.97–1.18)	
II	1.10 (1.02–1.19)	
III	1.18 (1.04–1.34)	
IV	0.97 (0.61–1.54)	
Asthma/COPD	1.63 (1.50–1.78)	<0.0001
Atrial fibrillation/flutter	1.35 (1.23–1.48)	<0.0001
BMI (per 2-unit increase)	1.03 (1.02–1.04)	<0.0001
Current smoker	1.37 (1.27–1.47)	<0.0001
Diabetes	1.54 (1.47–1.63)	<0.0001
Dyslipidemia	0.93 (0.88–0.98)	0.0101
Family history of premature CAD	0.94 (0.90–0.99)	0.026
Diastolic blood pressure (per 10-mmHg increase)	1.31 (1.28–1.34)	<0.0001
Current evidence of myocardial ischemia	1.19 (1.11–1.27)	<0.0001
Prior hospitalization for CHF	1.19 (1.06–1.32)	0.0023
Coronary artery bypass graft	0.93 (0.87–0.99)	0.025
Percutaneous coronary intervention	0.89 (0.84–0.95)	0.0001
Alcohol intake (units/week)		0.0005
>40	1.02 (0.69–1.51)	
20–40	1.10 (0.97–1.26)	
>0 and <20	0.91 (0.87–0.96)	
Physical activity level		<0.0001
Light physical activity most weeks	0.88 (0.82–0.94)	
≥20 min vigorous physical activity 1–2 times a week	0.75 (0.69–0.81)	
≥20 min vigorous activity ≥3 times a week	0.64 (0.59–0.70)	
Non-invasive test performed	0.80 (0.76–0.85)	<0.0001
Coronary territories with stenosis >50%; coronary angiography not done	1.13 (1.04–1.22)	0.0036
Coronary territories with stenosis >50%; right coronary artery	0.94 (0.89–0.99)	0.014
Not taking HR-lowering drugs	1.52 (1.42–1.61)	<0.0001
Reimbursement of cardiovascular agents		0.026
None	1.02 (0.96–1.09)	–
Full	0.94 (0.89–0.995)	–

BMI, body mass index; CAD, coronary artery disease; CCS, Canadian Cardiovascular Society; CHF, congestive heart failure; CI, confidence interval; COPD, chronic obstructive pulmonary disease; HR, heart rate; OR, odds.

## Discussion

This analysis provides a description of HR among stable outpatients with CAD. In this population, the mean (SD) pulse HR was 68.3 (10.6) bpm. Despite the fact that three quarters of the CAD population received treatment with beta-blockers, nearly half of the population had HR≥70 bpm, an emerging prognostic threshold in CAD patients with angina [6], [8], [15], [16], [21], and only 27.9% of all patients with CAD had HR≤60 bpm. Even among patients on beta-blockers, the proportion with HR≥70 bpm was 41.1%. Also, among patients with anginal symptoms, only 22.1% achieved a HR≤60 bpm, despite the fact that stable angina guidelines recommend a target HR of 55–60 bpm in patients with angina on beta-blockers [22]. This is consistent with observations from the EuroHeart Survey on angina [23], in which 19% of patients had HR≤62 bpm. By multivariable analysis, there were many independent predictors of HR≥70 bpm, a large proportion of which are markers of a poor health status, such as higher blood pressure, presence of diabetes, dyslipidemia, increased alcohol intake, history of chronic heart failure, increased body mass and lack of physical exercise. Among other independent predictors of HR≥70 bpm, angina class and evidence of myocardial ischemia were also important and strong correlates of elevated HR, as was the lack of use of HR-lowering agents.

These findings have important clinical implications: HR remains elevated in a substantial proportion of patients. Elevated HR has been associated with worse clinical outcomes in prior studies [4], [5], [6], [7], [8], [10]; and in the present study, was independently associated with more frequent evidence of myocardial ischemia and a worse anginal status. Elevated HR was, as expected, also more frequent among patients who did not receive HR-lowering agents. Thus, further HR reduction can be achieved and may yield substantial clinical benefit in these patients. Indeed, HR lowering with beta-blockers has been shown to have potent anti-ischemic and anti-anginal effects in patients with CAD [24] and yields improved clinical outcomes after myocardial infarction [11], but the effects of beta-blockers on HR are difficult to separate from their other major pharmacodynamic properties. Extrapolating from the evidence of prognostic benefit of beta-blockers in patients with angina who have a history of prior myocardial infarction (most of which antedate the advent of modern reperfusion therapy) or heart failure, both European and American guidelines for the management of stable angina suggest that beta-blockers be the first-line antianginal therapy in patients without contraindications [24], [25]. Yet, in the present analysis, poorly controlled HR was independently associated with diabetes mellitus, more severe angina class, higher blood pressure, evidence of myocardial ischemia, and physical inactivity, factors that point to patient populations most likely to benefit from beta-blockers. Because low blood pressure may be a limiting factor for using beta-blockers, it is noteworthy that patients not on beta-blockers did not actually have lower blood pressure. In fact, there was an association between increasing HR and higher mean systolic and diastolic blood pressures, which suggests that in this cohort, low blood pressure was not a major barrier to using beta-blockers and achieving HR control. In contradistinction to heart failure [26], there is no recommended target dose or beta-blocker molecule recommended in treating angina, and thus a wide variety of agents and doses are commonly used. Titration is usually based on resting HR achieved and therefore it is conceivable that increasing beta-blocker dosage might achieve superior HR control.

There are multiple potential barriers to more widespread use of beta-blockers at appropriate doses to achieve adequate HR control, such as inadequate knowledge of evidence or treatment goals by clinicians [27], access to care and reimbursement, co-morbidities that represent contraindications or decrease tolerance to beta-blockers, side effects of beta-blockers, and marketing efforts for other agents. Interestingly, in the present cohort, the proportion of patients fully reimbursed for drugs was actually greater among patients not receiving than among patients receiving beta-blockers, suggesting that lack of full reimbursement is not a major barrier to the prescription of beta-blockers. Likewise, it is striking that two thirds of patients not receiving beta-blockers had no apparent symptoms or conditions that would potentially contraindicate their use. Therefore, it is likely that it is possible to improve HR control by increasing the use of beta-blockers (and possibly their dose).

Whether other antianginal agents that lower HR might provide similar benefits as those of beta-blockers (i.e. beyond symptom control) is still debated. The rates of cardiac death and myocardial infarction are not different when comparing beta-blockers and calcium antagonists [28]. Pure HR-reducing agents such as ivabradine have been shown to be potent anti-anginal agents, alone and in combination with beta-blockers [12], [14]. In the BEAUTIFUL randomized trial in patients with stable CAD and left ventricular dysfunction [15], ivabradine did not improve clinical outcomes overall, but in a prespecified analysis, it reduced the incidence of coronary outcomes in the subset of patients with HR≥70 bpm [8]. These effects were more marked in a post-hoc analysis of patients with limiting anginal symptoms at baseline [16]. Whether pure HR reduction improves clinical outcomes in patients with CAD and without heart failure is currently being explored in the ongoing SIGNIFY randomized trial (http://www.controlled-trials.com/ISRCTN61576291). Also, because increased heart rate was correlated to an overall poor health status and lifestyle (e.g. with less physical activity, increased alcohol intake and higher prevalence of risk factors), lifestyle changes and correction of risk factors may be safe and effective ways to address the risk associated with increased heart rate.

The CLARIFY registry also provides a useful description of clinical characteristics, demographics, risk factors, drug treatment, and management of patients with CAD in stable outpatients from a broad geographic perspective. It provides a useful reference, which differs from the highly selected patient populations often enrolled in randomized trials [29], [30], and stems from a more diverse geographic representation, as trials are often skewed towards predominant representation of Europe and North America, and with diverse ethnicity.

Finally, while HR is emerging as a potentially important prognostic determinant in patients with CAD [4], [5], [6], [7], [8], [10] and heart failure [9], the determinants of HR are not well known. The present study allowed a detailed univariate and multivariable analysis of the correlates of elevated HR, which highlighted important and previously unrecognized factors in determining HR in this population. Apart from the previously mentioned greater burden of risk factors and markers of a poorer health status, such as increased alcohol intake, increased body mass index and lower level of physical activity, the presence of asthma/COPD, for instance, was one of the strongest predictors of an elevated HR (≥70 bpm) (odds ratio 1.63; 95% confidence interval 1.50–1.78, *p*<0.0001), which is consistent with the fact that use of beta-blockers is far less frequent in this population, who may in fact often receive beta agonists. In CLARIFY, 75.1% of the overall population received beta-blocker therapy (regardless of the dose) whereas this proportion was only 50.9% among patients with asthma/COPD. Likewise, increasing physical activity was associated with decreasing risks of elevated HR, a finding consistent with the well-documented effects of regular exercise on lowering HR [31].

While the CLARIFY registry is a large, international initiative taking place in 45 countries in four continents, it is subject to limitations. The study population may not fully reflect regional differences in clinical characteristics and patterns of care of stable CAD patients, and results may not therefore be representative of practice elsewhere. For example, no patients from the United States participated in CLARIFY. In addition, despite attempts to optimize the representativeness of the registry, it was not population-based. Finally, pending the availability of outcomes, the cross-sectional nature of the analysis limits the ability to draw causal inferences from the observations made which are susceptible to confounding.

In conclusion, despite the use of beta-blockers in three quarters of patients, nearly half of stable outpatients with CAD had a resting HR≥70 bpm. Even among patients on beta-blockers, 41% had HR≥70 bpm. An increasing HR was an independent correlate, among many other factors suggestive of an overall poorer health status, of higher prevalence and severity of angina, and higher prevalence of myocardial ischemia. These findings suggest that further HR lowering is possible in patients with stable CAD. Whether it will impact symptoms, ischemia, and risk of cardiovascular events is being tested.

## Supporting Information

Table S1 Baseline characteristics of the study population classified according to resting HR by palpation.
(DOCX)

Table S2 Characteristics of study population classified according to beta-blocker usage.
(DOCX)

Appendix S1 CLARIFY Registry Investigators.
(DOC)
