# Supplementary material for: Heart Rate and Use of Beta-Blockers in Stable Outpatients with Coronary Artery Disease
Source: PLoS One. 2012 May 3;7(5):e36284. doi: 10.1371/journal.pone.0036284 (PMC3343045; doi:10.1371/journal.pone.0036284)
Supplement: Table S1 — Baseline characteristics of the study population classified according to resting HR by palpation. (DOCX) [file pone.0036284.s001.docx]

**Table S1.** Baseline characteristics of the study population classified according to resting HR by palpation

| Variable | Patients with Data | Total Population  (*n* = 33,177) | Population According to palpation HR | | | *p*-Value |
| --- | --- | --- | --- | --- | --- | --- |
|  |  |  | **≤60 bpm**  **(*n* = 9,246)** | **61–69 bpm**  **(*n* = 9,322)** | **≥70 bpm**  **(*n* = 14,609)** |  |
| Age (y), mean (SD) | 33,150 | 64.2 (10.5) | 65.4 (10.1) | 64.1 (10.5) | 63.4 (10.6) | <0.0001 |
| Men, *n* (%) | 33,161 | 25,691 (77.5) | 7,533 (81.5) | 7,212 (77.4) | 10,946 (75.0) | <0.0001 |
| BMI (kg/m^2^), median (IQR) | 33,133 | 27.3 (24.8–30.3) | 27.1 (24.8–29.8) | 27.2 (24.8–30.1) | 27.4 (24.8–30.7) | <0.0001 |
| Ethnicity, *n* (%) | 33,177 |  |  |  |  | <0.0001 |
| Western descent |  | 21,323 (64.3) | 6,336 (68.5) | 6,086 (65.3) | 8,901 (60.9) |  |
| Black/African |  | 359 (1.1) | 103 (1.1) | 99 (1.1) | 157 (1.1) |  |
| Chinese |  | 2,809 (8.5) | 445 (4.8) | 834 (9.0) | 1,530 (10.5) |  |
| Hispanic |  | 1,621 (4.9) | 371 (4.0) | 439 (4.7) | 811 (5.6) |  |
| Japanese/Korean |  | 1,035 (3.1) | 187 (2.0) | 303 (3.3) | 545 (3.7) |  |
| South Asian |  | 2,713 (8.2) | 468 (5.1) | 636 (6.8) | 1,609 (11.0) |  |
| Unknown |  | 3,317 (10.0) | 1,336 (14.5) | 925 (9.9) | 1,056 (7.2) |  |
| Medical history, *n* (%) |  |  |  |  |  |  |
| Myocardial infarction | 33,170 | 19,788 (59.7) | 5,418 (58.6) | 5,632 (60.4) | 8738 (59.8) | 0.035 |
| PCI | 33,167 | 19,463 (58.7) | 5,673 (61.4) | 5,568 (59.8) | 8,222 (56.3) | <0.0001 |
| CABG | 33,164 | 7,769 (23.4) | 2,417 (26.2) | 2,228 (23.9) | 3,124 (21.4) | <0.0001 |
| Internal cardiac defibrillator | 33,170 | 408 (1.2) | 139 (1.5) | 110 (1.2) | 159 (1.1) | 0.016 |
| Pacemaker | 33,169 | 794 (2.4) | 232 (2.5) | 208 (2.2) | 354 (2.4) | 0.44 |
| Hospitalization for CHF | 33,169 | 1,555 (4.7) | 344 (3.7) | 402 (4.3) | 809 (5.5) | <0.0001 |
| Stroke | 33,169 | 1,323 (4.0) | 331 (3.6) | 338 (3.6) | 654 (4.5) | 0.0003 |
| Atrial fibrillation/flutter | 33,169 | 2,320 (7.0) | 670 (7.3) | 549 (5.9) | 1,101 (7.5) | <0.0001 |
| Asthma/COPD | 33,168 | 2,442 (7.4) | 530 (5.7) | 576 (6.2) | 1,336 (9.2) | <0.0001 |
| Risk factors and lifestyle, *n* (%) |  |  |  |  |  |  |
| Family history of premature CAD^a^ | 33,162 | 9,450 (28.5) | 2,743 (29.7) | 2,632 (28.3) | 4,075 (27.9) | 0.011 |
| Treated hypertension | 33,167 | 23,544 (71.0) | 6,299 (68.2) | 6,563 (70.4) | 10,682 (73.1) | <0.0001 |
| Diabetes | 33,165 | 9,680 (29.2) | 2,066 (22.4) | 2,600 (27.9) | 5,014 (34.3) | <0.0001 |
| Dyslipidemia | 33,169 | 24,837 (74.9) | 7,059 (76.4) | 7,021 (75.3) | 10,757 (73.7) | <0.0001 |
| Peripheral arterial disease | 33,166 | 3,242 (9.8) | 856 (9.3) | 902 (9.7) | 1,484 (10.2) | 0.068 |
| Smoking status | 33,168 |  |  |  |  | <0.0001 |
| Current |  | 4,115 (12.4) | 839 (9.1) | 1,099 (11.8) | 2,177 (14.9) |  |
| Former |  | 15,084 (45.5) | 4,697 (50.8) | 4,267 (45.8) | 6,120 (41.9) |  |
| Never |  | 13,969 (42.1) | 3,708 (40.1) | 3,951 (42.4) | 6,310 (43.2) |  |
| Alcohol intake (drinks/week), *n* (%) | 33,160 |  |  |  |  | <0.0001 |
| 0 |  | 16,051 (48.4) | 3,894 (42.1) | 4,497 (48.3) | 7,660 (52.5) |  |
| >0 and <20 |  | 15,923 (48.0) | 5,031 (54.4) | 4,500 (48.3) | 6,392 (43.8) |  |
| 20–40 |  | 1,073 (3.2) | 288 (3.1) | 290 (3.1) | 495 (3.4) |  |
| >40 |  | 113 (0.3) | 28 (0.3) | 30 (0.3) | 55 (0.4) |  |
| Stimulant drinks consumed, *n* (%) | 33,128 |  |  |  |  | <0.0001 |
| Coffee |  | 15,601 (47.1) | 4,833 (52.3) | 4,327 (46.5) | 6,441 (44.2) |  |
| Tea |  | 10,179 (30.7) | 2,359 (25.5) | 2,952 (31.7) | 4,868 (33.4) |  |
| Neither |  | 7,348 (22.2) | 2,043 (22.1) | 2,031 (21.8) | 3,274 (22.5) |  |
| Daily intake of stimulant drinks (cups/day), median (IQR) | 25,720 | 2.0 (2.0–4.0) | 2.0 (2.0–4.0) | 2.0 (2.0–4.0) | 2.0 (2.0–4.0) | <0.0001 |
| Physical activity, *n* (%) | 33,157 |  |  |  |  | <0.0001 |
| None |  | 5,403 (16.3) | 1,236 (13.4) | 1,458 (15.7) | 2,709 (18.6) |  |
| Light physical activity most weeks |  | 17,029 (51.4) | 4,307 (46.6) | 4,849 (52.0) | 7,873 (53.9) |  |
| ≥20 min vigorous physical activity 1–2 times/week |  | 5,558 (16.8) | 1,792 (19.4) | 1,582 (17.0) | 2,184 (15.0) |  |
| ≥20 min vigorous physical activity ≥3 times/week |  | 5,167 (15.6) | 1,906 (20.6) | 1,428 (15.3) | 1,833 (12.6) |  |
| Angina and CCS class, *n* (%) | 33,168 |  |  |  |  | <0.0001 |
| No angina |  | 25,863 (78.0) | 7,634 (82.6) | 7,276 (78.1) | 10,953 (75.0) |  |
| Angina Class I |  | 2,088 (6.3) | 512 (5.5) | 609 (6.5) | 967 (6.6) |  |
| Angina Class II |  | 3,878 (11.7) | 844 (9.1) | 1,084 (11.6) | 1,950 (13.4) |  |
| Angina Class III |  | 1,257 (3.8) | 232 (2.5) | 329 (3.5) | 696 (4.8) |  |
| Angina Class IV |  | 78 (0.2) | 23 (0.3) | 18 (0.2) | 37 (0.3) |  |
| CHF symptoms including NYHA class | 33,163 |  |  |  |  | <0.0001 |
| No CHF |  | 28,229 (85.1) | 8,239 (89.1) | 7,881 (84.6) | 12,109 (82.9) |  |
| CHF Class II |  | 4,131 (12.5) | 873 (9.4) | 1,220 (13.1) | 2,038 (14.0) |  |
| CHF Class III |  | 803 (2.4) | 132 (1.4) | 218 (2.3) | 453 (3.1) |  |
| Creatinine (mmol/l), median (IQR) | 25,054 | 0.09 (0.08–0.10) | 0.09 (0.08–0.10) | 0.09 (0.08–0.10) | 0.09 (0.08–0.10) | 0.0004 |
| Hemoglobin (mmol/l), median (IQR) | 22,250 | 8.69 (8.07–9.31) | 8.75 (8.19–9.31) | 8.69 (8.07–9.31) | 8.69 (8.01–9.31) | <0.0001 |
| HR palpation (bpm), mean (SD) | 33,177 | 68.3 (10.6) | 56.6 (4.1) | 65.2 (2.2) | 77.7 (7.8) | – |
| HR ECG (bpm), mean (SD) | 24,593 | 67.2 (11.4) | 57.0 (6.6) | 64.9 (6.4) | 75.8 (10.0) | – |
| SBP (mmHg), mean (SD) | 33,173 | 131.0 (16.7) | 129.7 (16.3) | 130.2 (16.1) | 132.4 (17.2) | <0.0001 |
| DBP (mmHg), mean (SD) | 33,173 | 77.2 (10.0) | 75.4 (10.0) | 76.7 (9.7) | 78.8 (10.2) | <0.0001 |
| LVEF (%), mean (SD) | 22,795 | 56.1 (11.1) | 57.2 (10.6) | 56.5 (10.8) | 55.1 (11.5) | <0.0001 |
| Non-invasive test for myocardial ischemia^b^, *n* (%) | 33,145 | 20,514 (61.9) | 6,496 (70.3) | 5,806 (62.4) | 8,212 (56.3) | <0.0001 |
| Current evidence of myocardial ischemia, *n* (%) | 33,145 | 5,363 (16.2) | 1,357 (14.7) | 1,499 (16.1) | 2,507 (17.2) | <0.0001 |
| ECG rhythm, *n* (%) | 24,552 |  |  |  |  | <0.0001 |
| Sinus rhythm |  | 23,315 (95.0) | 7,014 (96.1) | 6,557 (96.1) | 9,744 (93.4) |  |
| Atrial fibrillation/flutter |  | 837 (3.4) | 142 (2.0) | 173 (2.5) | 522 (5.0) |  |
| Paced rhythm |  | 400 (1.6) | 141 (1.9) | 91 (1.3) | 168 (1.6) |  |
| LBBB, *n* (%) | 24,576 | 1,200 (4.9) | 319 (4.4) | 342 (5.0) | 539 (5.2) | 0.045 |

BMI, body mass index; CABG, coronary artery bypass graft; CAD, coronary artery disease; CCS, Canadian Cardiovascular Society; CHF, congestive heart failure; COPD, chronic obstructive pulmonary disease; Cx, circumflex; DBP, diastolic blood pressure; ECG, electrocardiogram; HR, heart rate; IQR, interquartile range; LAD, left anterior descending; LDL, low-density lipoprotein; LBBB, left bundle branch block; LVEF, left ventricular ejection fraction; NYHA, New York Heart Association; PCI, percutaneous coronary intervention; RCA, right coronary artery; SBP, systolic blood pressure; SD, standard deviation.

^a^MI, sudden death, stable angina at age <55 years (men) or <65 years (women) in a first-degree relative.

^b^Stress ECG, stress echocardiography, myocardial imaging.
