# Supplementary material for: Heart Rate and Use of Beta-Blockers in Stable Outpatients with Coronary Artery Disease
Source: PLoS One. 2012 May 3;7(5):e36284. doi: 10.1371/journal.pone.0036284 (PMC3343045; doi:10.1371/journal.pone.0036284)
Supplement: Appendix S1 — CLARIFY Registry Investigators. (DOC) [file pone.0036284.s003.doc]

**Appendix.** CLARIFY Registry Investigators

**Executive Committee**: P G Steg (chair), Paris, France; R Ferrari, Ferrara, Italy; I Ford, Glasgow, UK; K Fox, London, UK; J C Tardif, Montreal, Canada; M Tendera; Katowice, Poland.

**Steering Committee**: *Argentina*: Prof. Dr. Fernando José Sokn; *Australia:* Prof. Christopher Reid; *Austria:* Prof. Irene Lang; *Belgium*: Dr. Frank Van den Branden; *Brazil*: Prof. Luis Machado César; Prof. Marco Antonio Mattos; *Brunei*: Dr. Hj. Nazar Luqman; *Bulgaria*: Prof. Assen Goudev; *Canada:* Prof. Paul Dorian; *China*: Prof. Dayi Hu; *Czech Republic*: Prof. Petr Widimsky; *Denmark:* Dr. Christian Hassager; *France*: Prof. Nicolas Danchin; *Germany:* Prof. Dr. med. Stefan Kääb; *Greece:* Prof. Panos Vardas; *Gulf Countries:* Dr. Kadhim J. Sulaiman (*Oman*), Dr. Wael Al Mahmeed (*UAE*), Dr. Jassim Al Suwaidi (*Qatar*), Dr. Ibrahim Al Rashdan (*Kuwait*), Dr. Fuad Abdulkader (*Bahrain*); *Hungary:* Prof. Béla Merkely; *India*: Prof. Upendra Kaul; *Ireland*: Prof. Kieran Daly; *Italy*: Prof. Luigi Tavazzi, Prof. Roberto Ferrari; *Korea*: Prof. Yangsoo Jang; *Latvia*: Prof. Andrejs Erglis; *Lithuania:* Prof. Aleksandras Laucevičius; *Malaysia*: Dr. Ahmad Nizar Jamaluddin; *Mexico:* Prof. Marco Alcocer Gamba; *Netherlands*: Dr. Igor I. Tulevski; *Poland*: Prof. Janina Stępińska; *Portugal*: Dr. Joao Morais; *Romania*: Prof. Dr. Cezar Macarie; *Russia*: Prof Rafael Oganov, Prof Svetlana Shalnova; *Saudi Arabia*: Prof Muayed Al-Zaibag; *Singapore:* Dr. Mak Koon Hou; *Slovakia:* Assoc. Prof. Gabriel Kamensky; *Slovenia*: Prof. Zlatko Fras, Dr. Vojko Kanič; *South Africa:* Prof. Datshana Prakesh Naidoo; *Spain:* Prof. José Luis Zamorano; *Switzerland*: Prof. Hans Rickli, Dr. Andres Jaussi; *Thailand*: Assco. Prof. Charn Sriratanasathavorn; *UK*: Dr. Paul Kalra; *Ukraine*: Prof. Mykhailo Lutai, Prof. Oleksandr; *Vietnam*: Prof Lan Viet Nguyen; *West* Indies: Dr. Ronald Henry.

**CLARIFY Investigators**:

*Argentina:* A. Ahuad Guerrero, M. Basara, F. Belcastro, J.A. Bertarini, C. Cazenave, H. Dreycopp, J. Egido, J. Estrella, D. Garofalo, J. Giordano, H. Lagioia, N. Lago, R. La Greca, L. Lema, N. Lopez Cabanillas, H. Luquez, C. Miller, E. Prada, P. Rodenas, R.G. Schena, G. Suarez, A. Tomatti; *Australia*: D. M. Colquhoun, A. Conradie, S. Cox, D. Cross, R. Fathi, B. Fitzgerald, I. Hamilton-Craig, G. Holt, S. R. Jayasinghe, N. Mai, J. Moolman, R. A. Motyer, K. Phillips, A. Rafter, A. Rahman, A. Rainbird, G. Scalia, A. Taylor, P. West, K. Alford, R. Amor, P. Astridge, B. Bastian, F. Bates, M. M. Doohan, J. Du Plooy, J. C. Ford, L. Kanagaratnam, V. Khoury, R. Parkin, J. Rogers, G. Sceats, A. Waldman, D. Wang, S. Wright, J. Ardill, P. Aylward, J. F. Beltrame, J. Bradley, W. Heddle, M. Joseph, S. Rajendran, S. Varughese, E. Brice, B. Hockings, J. Janssen, A. Kozlowski, J. O'Shea, D. A. Playford, K. Woollard, A. Ajani, G. Barron, N. Better, B. Chan, R. Chan, J. Cotroneo, J. T. Counsell, D. S. Eccleston, B. H. R. Forge, A. Hamer, M. Horrigan, V. M. J. Jelinek, R. Lew, D. O'Donnell, F. Panetta, M. Sebastian, A. Soward, P. Srivastava, N. F. Strathmore, S. Sylivris, G. Szto, V. Veth, T. Yip; *Austria*: R. Badr-Eslam, L. Kleemann, G. Steurer, B. Mörz-Proszowski, F. Auhser, U. Teleky, G. Sepp, A. Beinhauer, D. Kerö, C. Lavicka, T. Perger, V. Hadjiivanov, M. Feldner-Busztin, R. Mika, W. Filip, A. Mahr, J. Toplak, M. G. Millauer, P. Haralambus, K. Walcher, K.H. Karner, E. Ziak, P. Painsipp, U. Frank, A. Suntinger, W. Gritsch, G. Bode, R. Herrmann, R. Raffelsberger, H. Topf, E. Moser, J. Föchterle, T. Honsig, K. Mayr, H. Mayr, R. Kaserbacher, A. Dzien, E. Galehr, M. Felbermayer, R. Schwarz; *Belgium*: R. Amini, H. Appeltants, A. Ballet, J-P. Bar, J. Beckers, J-M. Bergen, G. Berkenboom, X. Bernard, T. Bouvy, R. Briki, M. Claeys, Y. Dascotte, L. Davin, T. De Backer, F. De Keyser, A. De Meester, S. De Ridder, P. Dendale, K. Denef, E. Dhondt, M. Emonts, J. T. M. Geraedts, M. Goethals, J-M. Grégoire, E. Haine, T. Herbots, E. Hoffer, W. H. J. Hutse, A. Kassab, P. Lafontaine, P. Lancellotti, P. Lefebvre, H. Lesseliers, A. Lozano, R. Maamar, C. Martinez, J-F. Noël, G. Odent, A. Pasquet, B. Peperstraete, P. Purnode, A. Rogowsky, M. Rosseel, J-P. Salembier, P. Surmont, P. Thermol, A. M. F. Vandeplas, S. Van de Walle, F. Van den Branden, P. Vandergoten, B. G. Vanhauwaert, L. Vanneste, J. Vercammen, D. Verleyen, D. Vermander, G. Vervoort, C. Weytjens, N. Yanni; *Brazil*: A. da Costa Pereira, A. Rocha de Lorenzo, A. Felice Castro Issa, B. Mahler Mioto, C. de Brito Vianna, C. A. W. Segre, C. J. Grupi, C. Okawabata, D. Favarato, E. Giusti Rossi, F. Fernandes, F. Pitella, F. J. Alvarez Ramires, F. Henpin Yue Cesena, J. F. Monteiro Ferreira, J. F. Junior, L. Tonet, L. Nastari, L. Machado Cesar, L. H. Gowdak, M. A. Matos, M. Moretti, P. C. Morgado, R. Vicente Amato, R, Tadeu Munhoz, S. R. Coimbra; *Brunei*: H. N. Luqman - *Bulgaria*: S. Yakovova, M. Mantcheva, V. Mincheva, L. Baurenski, K. Karastanev, V. Yordanova, Y. Peneva; *Canada*: A. Bailey, P. Wong, M. Fagan, G. Sabe-Affaki, F. M. Villasenor, P. Belisle, W. K. Son, D. E. Manyari, N. Giacomantonio, B. J. Lubelsky, D. Ezekiel, J. C.S. Leong, A. Grover, J. Vavougios, Y. Pesant, A. M. Kushner, M. M M. Yeung, G. E. Vertes, F. J. Nasser-Sharif, A. H. K. Abdulla, D. Spensieri, A. Roy, T. T. Nguyen, M. Leclair, P. Morra, C. Everton Biglow, J. F. Baril, K. Lai, D. S. Wong, V. Martinho, G. A. Antoniadis, G. R. Searles, D. Rouse, G. Brisson, S. King Wong, R. S. Collette, M. S. C. Ho, C. Constance, R. Gendreau, G. W. Kellam, T. A. Cieza Lara, H. A. Boyrazian, M. Shamsuzzaman, D. R. Spink JR, A. P. T.Wong, R. S. Grewal, C. Che, J. Janes, N. Hechtenthal, M. Czarnecka, D. Saulnier, G. Levesque, P. F. Clavette, D. R. Kennedy, A. Kokis, T. L. Orenstein-Lyall, A. Shekhar Pandey, J. Robb, G. Verret, W. Czarnecki, W. W. H. Tsui, F. Perreault, G. Chouinard, G. Lafrance, G. M. Fullerton, J. P. Lavoie, P. LeBouthillier, Q. H. Tran, I. Rodriguez Marrero, F. B. Ramadan, P. Talbot, M. A. Fazil, J. Yi-Ming Cha, S. Garg, R. Chehayeb, B. Roy, Y. K. Chan, H. E. Harlos, H. B. Matheson, R. Patel, G. F. Vaz, J. S. Bhatt, E. Liu, T. H. Ashton, H. Sullivan, L. P. Quinn, K. Yared, A. Gupta, B. Sullivan, J. Campbell, S. Pallie, H. Kim, S. Vizel, D. Savard, J. M. Cherry, J. Gold, S. Chiu, G. Brouillette, R. R. Singh, S. Varma, A. Belanger, J. L. Myburgh, J. Berlingieri, W. Nisker, G. Boutros, A. I. Bakbak, W. Healley, L. Lasalle; *China*: F. Liu, C.Tu, S. Lv, X. Liu, H. Gao, H. Li, H. Zhao, L. Cao, S. Zhao, Y. Wang, D. Wu, F. Gu, G. Pan, P. Liu, X. Wang, H. Jiang, J. Li, J. Wang, L. Zhang, X. Wang, Y. Ke, D. Li, G. Chen, H. Xue, Q. Jin, W. Dong, Y. Chen, Z. Fu, J. Wang, H. Hu, Q. Liang, X. Yang, Z. Zhou, Z. Xu, C. Shao, H. Zhang, H. Pei, L. Song, M. Yu, T. Guan, Y. Tang, Y. Wu, M. Yang, Q. Ceng, X. Chen, L. Lin, Y. Peng, X. Yan, E. Yao, X. Zheng, B. Chen, H. Chen, W. Chen, R. Wang, Y. Zheng, H. Tan, S. Zhou, Y. Zhou, Z. Liu, R. Wang, Q. Lu, L. Lai, J. Pan, L. Wang, Q. Fu, J. Peng, N. Du, H. Li, Y. Lv, W. Miao, H. Wang, Y. Pu, T. Wang, M. Dong, L. Gong, X. Liu, J. Zhang, L. Zhang, Z. Chen, Q. Jiang, F. Ma, W. Xu, M. Dai, Y. Wang, J. Wu, X. Yu, C. Chen, Y. Huo, L. Sun, W. Gao, X. Liu, Z. Li, Y. Hu, H. Li, M. Chen, G. Li, M. Xue, Y. Yao, X. Pan, Z. Sang, G. Zhao, J. Pan, J. Hang, L. Sun, S. Ma, G. Zhang, G. Zhou, W. Li, Y. Wang, B. Zhu, B. Yu, H. Wang, S. Zhu, X. Chen, H. Zhang, J. Mao, M. Xu, Q. Liu, Q. Huang, Y. Xie, L. Feng, F. Chen, L. Chen, Y. Liu, X. Pei, A. Sun, Z. Tian, W. Wang, H. Yang, X. Yang, A. Yu, M. Zhang, C. Zhang, X. Guan, X. Zhou, Y. Li, Y. Xing, K. Chen, L. Luo, S. Dong, Y. Chen, Y. Zhou, S. Zhou, Y. Zhang, F. Ai, G. Chen, C. Xiong, F. Yang, K. Yang, Y. Zhou, J. Yan, M. Zhu, A. Zhang, G. Shan, J. Chen, J. Guo, S. Wu, Z. Li, J. Chen, G. Shan, Z. Li, S. Wu, A. Zhang, L. Li, R. Liu, Y. Yang, Y. Yang, H. Zhang, G. Chen, X. Gao, Z. Du, L. Liang, Y. Wu, Y. Zhang, Y. Zhao, J. Qian, L. He, L. Xiong, P. Chen, P. Chen, L. He, L. Xiong, C. Peng, J. Zhu, J. Liu, X. Xie, F. Jiang, A. Li, J. Li, Q. Yang, M. Chen, H. Cong, Y. Guo, N. Ren, J. Xiao, Y. Zhang, R. Zhao, J. Jiang, X. Chen, X. Deng, L. Li, L. Wang, S. Wang, K. Wu, X. Zhang, W. Du, D. Shuang, J. Wei, C. Yuan, F. Li, X. Ou, Y. Ou, G. Yu, S. Zhang, J. Gao, Z. Qian, G. Wu, S. Zheng, D. Xu, J. Xie, W. Ren, X. Yao, Y. Wang, Z. Chen, B. Cai, L. Li, J. Lv, F. Li, J. Dong, J. Li, L. Feng, Z. Deng; *Czech Republic*: J. Bozkova, J. Carda, S. Dedkova, A. Dufka, J. Fridrich, T. Hodac, R. Jirmar, A. Kadleckova, M. Karlicek, J. Krupicka, J. Kuchar, V. Lavicka, J. Leso, Z. Lorenc, M. Micko, P. Navratil, I. Petrova, P. Povolna, L. Raisova, P. Raska, V. Ravlyk, S. Schlesingerova, E. Smrckova, P. Sternthal, H. Stursova, P. Vymetal, L. Zaoral; *Denmark*: P. Wiggers,, J. Markenvard, L. K. Andersen, L. Frost, J. Refsgaard, S. Strange, K. Egstrup, R. Sykulski, P. Hildebrant, T. Haghfelt, M. Ege; *France*: S. Cattan, M. Adam-Blanpain, M. Adda, N. Aimouch, L. Ardouin, S. Assouline,A.Aumjaud, C. Barjhoux, R. Baroudi, C. Beaurain , M. A. Bennouna, A. Bernard, C. Bernardeau, E. Blanc, I. Blum-Decary, G. Bodur, C. Boesch, J. Bonal, R. Bonhomme, J. L. Bonnet, J. Bories, M. L. Bourachot, F. Brumelot, M. Brunehaut Petaut, C. Brunschwig, P. Buffet, P. Calmettes, I. Centa, B. Chartier, P. Chemin, F. Chometon, J. Cohen, R. Colin, Y. Cottin, F. Crespo, A. Dabboura, F. David, P. Dehayes, P. Dematteo, O. Dibon, P. Dodemant, V. Dormagen, X. Dreyfus, J. M. Dubois, F, Duclos, M. Ducoudre, O. DUPREZ, P. Durand, E. Durand, P. Egloff, M. Escande, M. C. Escourrou Berdou, G. Esna Ashari, I Feldmann, J. Ferrieres, E. Foltzer, B. Fontanet, M. Garandeau, T. Garban, S. Geffroy, T. Gillet, S. Godart, P. Gosse, P. Gratia, O. Greiner, A. Gueusquin, E. Guiu, J. M. Guy, S. Haddad, V. Hennebelle, S. Honorat, A. Hourany, G. Hua, P. Jacquier, S. Jean, R. Jeremiasz, P. Kohler, A. Lacroix, M. Leandri, Y. Lemiere, M. Liautard, P Loheac, J. C. Louchart, P Magnus, B, Maheu, H. R. Malaterre, G. Manchet, J. Mantoux, D.Manzi, M. Marachli, M. Maroun, N. Meneveau, E. Messas, J. L. Mougeolle, T. Mouhat, J, J. Muller, M. Naisseh, P. Nocon, D. Onger, A. Ouguoujil, M. Ovize, E. Page, K. Pareathumby, A. Pleskof, P. Poinson, G. Pons, P. Pouderou, J. N. Poujois, V. Probst, F. Prunier, L. Prunier, V. Puel, D. Rechtman, R. Rennert, B. Rijavec, Y. Riou, J. Robert, C. Roche, G. Roul, B. Salaun, B. Saleh, A. Sandalian, M. Sander, A. Schenowitz, A. Silvestre, H. Soleille, S. Tabet, M. Tardy, F. Thomas-Richard, B. Truong, J. Varaldi, H Vial, J. M. Walch, M. Wazana, R. Zeitouni, H. Audibert, F. Alizon, A. Amlaiky, M. Asplanato, C. Baranes, M. Bariaud, F. Bernasconi, P. Bousquet, C. Ceraulo, G. De Geeter, J. Donetti, B. Doucet, J. Doucet, T. Dutoya, D. Ennouchi, M. H. Fallacher, G. Fouquet, V. Fourchard, J. Gdalia, G. Grollier, S. Guerard, P. A. Jeannerat, Y. Jobic, V. Joulie, P. Jourdain, V. Jouve, R. Ketelers, G. Khaznadar, P. Kohan, B. Koujan, B. Lammens, I. Landragin, E. Le Moal, D. M'Bey, F. Maes, S. Maheas Morlet, R. Massabie, D. Meddah, F. X. Meriaux, C. Mestre-Fernandes, P. Meyssonnier, M. Migliore, J. Milewski, J. F. Millet, S. Mingam, P. Nazeyrollas, F. Paganelli, F. Pellerin, F. Petitjean, A. Pinzani, A. Pladys, P. Primot, A. Pucheu, A. Rahali, P. Ravoala, D. Rousson, P. Samama, M. Sardon, R. Silvestri , P. Soskin, X. Tabone, C. Tricot, B. Vaquette, M. Vogel, M. Weingrod, V. Aboyans, R. Amoretti, J. Aubry, P. Berthezene, D. Binet, X. Bonnaud, P. Bonnet, A. Bonny, T. Bouchaya, C. Boureux, J. M. Bourgeois, L. Brottier, B. Cavert, S. Cleron, E. Dechoux, C. Delhomme, J. P. Detienne, J. P. Dubs, B. Faudon, F.Fellous, R. Fressonnet, Y. Garaud, D. Garcia, M. Geneves, J. L. Gleizes, C. Guyetand, B. Hermellin, D. Iovescu, J. P. Kanner, P. Khanoyan, A. Leherissier, A. Maximovitch, B. Merian, P. Messali, Y. Moreau, J. Moyal, L. Payot, L. Petoin Peuch, J. L. Prevot, P. Raymond,D. Relange, S.Reymond, J. F. Robert, H. Rosenstein, J. Schneider, R. Schultz, P. Tanielian, F. Thoin, L. Thomas, P. Touzet, G. Steg, G. Amiel Oster Sauvinet, F. Baylac Domengetroy, K. Chamou, B. Etcheverry, J.L. Farges, J. Y. Fraboulet, M. Goralski, D. Janody, B. Mamez, W. Manlay, F. Paillard, F. Pelier, A. Petit, M. Skonieczny, R. Augarde, J. B. Fournier, S. Liandrat, P. Lim, A. I. Noury, D. Paris, M. Saade, J. M. Stordeur, N. Danchin, M. Pornin, L. Fauchier, M. Galinier, M. A. Balice-Pasquinelli, P. Sosner, S. Yvorra, E. Delcoulx, F. Mouquet, J. E. Poulard, A. Sudre, P. Heno, F. Biausque, M. Guenoun, G. Attia, S. Pouwels, L. Carpentier, E. Verbrugge, C. Ziccarelli, M. Elkohen, J. Tricoire, P. Lang, O. Huttin; *Germany*: B-M. Altevogt, U. Altmann, M. Baar, S. Berrisch-Rahmel, A. Birkenhagen, I. Bläse, R. Blindt, R. Bosch, A. Brattström, H-H. Breuer, M. Castrucci, S. Cicek-Hartvig, R. Cierpka, M. Claus, M. Deissner, M. Drexler, T. Eggeling, G. Eisele, D. Enayat, S. Frickel, S. Gessner, K. Giokoglu, J. Gmehling, F. Goss, P. Grooterhorst, D B. Gysan, R. Haberl, W. Haerer, N. Hassler jun, S. Heinemann, F. Henschel, M. Hinrichsen, W. Hofer, A. Hofmeister, G.Hoh, E. Horstkotte, F. Jäger, M. Jeserich, U. Keil, H. Killat, S. Kimmel, M. Kindel, P. Kindler, S. Kleta, J. Könemann, K. König, H. Krause-Allmendinger, K. Kronberg, I. Kruck, V. Männl, A. Meinel, G. Mentz, E. Meyer-Michael, F. Mibach, S. Möller, S. Muth, E. Nelböck-Huber, D. Ohlmeyer, Z. Özkan-Rashed, C- P. Paulus, S. Perings, J. Placke, C. Raters, N. Reifart, A. Rink, K. Rybak, I. Salecker, K-H. Schermaul, E. Schmidt, K-H. Schmitz, N. Schön, T. Schröder, B. Sievers, M. Simon, U. Spengler, M. Speth-Nitschke, A. Stumpp, S. Szabo, J. Taggeselle, A. Tamm, A. Thelemann, C. Thelemann, H. Thümmel, G. Unger, A. Utech, J. Volmar, B. Wauer, G. Wehr, L. Weinrich, R. Weinrich, U. Windstetter, J H. Wirtz, N. Wittlich, P. Ziehn, P. Zündorf– *Gulf Countries*: *Oman*: Y. Al Wahshi, P. P. Singh, A. Narayan, F. Al Tamimi, J. Al Yazeedi, M. Ayche, A. Al Lawati, M. Al Dhanki, *United Arab Emirates -* A. Salustri, A. Al Sousi, T. Salah, M. Y. Tamimi, A. Agrawal, A. Wassef, F. Baslaib, G. Al Radaideh, A. Yusufali, N. Bazargani, *Kuwait -* M. Akbar, H. Abdel Wahab, S. Abdel Malak, .I. Ghaly, S. Al Ghool, F. Al Kandari, M. Haiba, M. Alanbaei, *Qatar -* A. El Menyar, M. M. Gomaa, *Bahrain -* A. Khalifa, T. Garadah; *Greece*: C.Avgerinos, O. Gouli, D. Stergiou, I. Alexopoulos, C. Pappas, I. Petropoulos, G. Chatzioakim, N, Pontikakis, C. Priftis, P. Mpompoth, I. Bourazanis, A Papathanasioy, S. Avlonitis, C. Zakopoulos, G. Koutsimpanis, I. Tsamopoulos, C. Christoforidis, V. Zachos, P. Kalaras, M. Karachaliou, C. Liatas, G. Pournaras, G. Theodorakis, I. Orestis, K. Panisois, E. Chalkiadakis, V. Arfaras;Melainis, G. Kolios, P. Boutsikos, A. Kotsalos, D. Mitropoulos, A. Samothrakitis, K. Svolis, E. Anastasiou, T. Gkinis, P. Dalampyras, A. Kalampalikis, I. Leontaridis, S. Gabriilidis , I. Konstantinidis, V. Plastiras, P. Tarenidis; *Hungary*: I. Marozsán, I. Édes, I. Czuriga, A. Cziráki, K. Tóth, Á. Dongó, P. Túri, T. Forster, J. Borbola, B. Bachmann, G. Masszi, M. Orbán, G. Gerges, G. Balogh, É. Bajcsi, M. Sereg, Cs. A. Dézsi, I. Takács, L. Nagy, B. Kisjós, A. Jánosi, A. Nagy, K. Nagy, A. Büttl, J. Lippai, Zs. Sziegl, Zs. Malkócs, A. Földi, K. Fikker, E. Szabó, Prof. B. Merkely; *India*: R. Gupta, S. Natarajan, J. Dalal, R. K. Saran, A. Mehta, M. P. Samal, I. A. Khan, T. Ghose, J. P. S. Sawhney, T. Roy, S. Chandra, S. Modi, M. M. Singh, G. Vijayaraghavan, L. Sreenivasa Murthy, S. S. Ramesh, Dr Dayasagar Rao V, M. S. Chenniappan, A. Vadavi, K. Kunhali, K. Srinivasa Reddy, Su. Thillai Vallal, P. Khera, A. Dasbiswas, K. Ganguly, S. S. Chatterjee, B. Prasad, D. Shukla, A. K. Trivedi, R. Ahuja, J. Deb, J. Rawal, R. Karnik, M.S. Hiremath, D. K. Kumbla, S. R. Shetty, N. S. Chonkar, Late M Juneja, B.K. Goyal– *Ireland:* R. Sheahan, N. Mulvihill, C. Vaughan, S. Fleming, P. Shiels, P. Keelan, T. Kiernan, J. Cosgrave, B. Day, K. Kelly, F. MacNamara, B. Maguire, A. Clifford, A. O'Gara; *Italy*: G. Guardigli, G. Riccioni, R. Pedretti, S. Felis, V. Pernice, A.Lillo, P. Gori, F. Zacà, F. Giacomazzi, P. Terrosu, C.Cernetti, R. Antonicelli, G. Ansalone, M. Balbi, C. Tamburino, S. Tantillo, F. Proietti, V. Mallamaci, D. d'Este, F. Silvestri, F. Magliari, N. Capuano, N. Marchionni, M. Turiel, P. Maxia, L. Marullo, A. Vicentini, G. Pes, G. Caridi, A. Grieco, B. Doronzo, A. Lacchè, F. Massari, S. Orazi, G. Antonelli, M. Provvidenza, A. Nicolino, S. De Servi, G. Sinicropi, G. Maragoni, P. Azzolini, E. Brscic, A. S. Bongo, G. Perna, B. Perna, C. La Rosa, E. Mossuti, R. Ferrante, M. E. Petrillo, M. Castellari, P. Di Pasquale, F. Saporito, F. Alitto, R. Testa; *Korea*: S. M. Kang, B. K. Koo, S. K. Hong, W. Kim, S. H. Lee, H. S. Seo, H. C. Gwon, D. H. Kang, H. M. Kwon, I. H. Chae, S. J. Oh, J. H. Shin, C. W. Goh, J. H. Zo, T. J. Hong, D. S. Kim, T. J. Cha, J. K. Ryu, Y. J. Kim, J. Y. Hwang, S. H. Hur, M. H. Jeong, S. K. Oh, D. K. Jin, K. T. Jung, J. Y. Rhew, S. Lee, D. W. Jeon, S. H. Kim; *Latvia*: I. Mintale, G. Latkovskis, S. Hansone, N. Rozkova, A. Baika, I. Jasinkevica, S. Abele, I. Laizane, N. Pontaga, V. Ecina, I. Mihailova, A. Kondratovica; *Lithuania*: A. Laucevičius, R. Jurgaitienė, R. Šlapikas, G. Barauskienė, E. Jankauskienė, S. Revienė, T. Vaišvila, D. Zaronskienė, O. B. Šlapikienė, N. Kupstytė, E. Rinkūnienė, R. Steponėnienė, J. Kojelienė, J. Badarienė, V. Dženkevičiūtė, E. Sadauskienė, I. Butkuvienė, R. Stankevičius, R. Paliulionienė, R. Snikytė, R. Mažutavičius; *Malaysia*: A. N. Jamaluddin, A. A. Abdul Rahim, Ah. K. Mohamed Yusof, K. H. Chee, A. Sadiq, S. Ramanaidu, K. H. Sim, T. K. Ong, A. Y. Y. Fong, B. C. Chang, S. K. Chua, Y. L. Cham, N. A. Mohd. Amin, T. K. Ong, S. K. Tan, K. Chandran, Y.W Cheah, J. Sinnadurai, C. K. Choor, K. K. Sia, C. C. Ang, J. Singh, M. Z. Abdul Wahab, A. K. Ghapar, A. Muthu, M. Kauthaman, A. H. Jaafar, K. H. Ng, A. R. Tahir, H. Abdul Manap, B. S. K. Ch'ng, E. T. Ch'ng, O. Ismail, A. S. Sahar, B. B. Abdul Kareem, S. K. Ma, H. B. Liew, R. K. M. Bhaskaran, R. P. Shah, K. L. Joseph, H. Noor Hasni, W. K. Ng, G. H. Choo, C. K. Yeo, V. M. Lai, Y. C. Lai, M. H. Tay, B. A. Lim; *Brunei*:H. N. Luqman*;* *Mexico*: Guillermo Llamas Esperon, J. de Jeús Zuñiga Sedano y America Alvarez, F. Azar Manzur, C. Jerjes Sánchez, J. Cerda Rojas, J. Carrillo Calvillo, F. Petersen Aranguren, C. Martínez Sánchez, G. Vieyra, S. González Romero, A. Puente Barragán, F. Redding Escalante, J. Chávez Paez, E. Fernandez Valadez, E. Gaxiola, L. E. Manautou, O. Henne Otero, M. Barrera Bustillos, J. L. Leyva Pons, E. Gómez Alvarez, J. R. Romo Santana, J. Martínez Redding, A. Arias Mendoza, I. Rodríguez Briones, J. de Jeús Rivera Arellano, J. L. Arenas León, M. Alcocer Gamba, E. Alexanderson, M. E. Ruíz Esparza,L. A. Elizondo Sifuentes, J. L. Briseño, S. Sandoval, A. Castro, R. Cue Carpio, E. Rodríguez, G. Rojas, G. Solache, R. Díaz, R. Baleón, C, Ferreyra Solorio, H. Alberto Ramírez Reyes, M. López Martínez, M. A. Romero Maldonado, J. Escobedo de la Peña, J. Hilario Jimenez Orozco, F. A. Reyes Cisneros, J. Alvarez Gil, G. Bautista, López, M. Odín de los Ríos Ibarra; *Netherlands*: I. I.Tulevski, G. A. Somsen, J. E. Wittekoek, K. Miedema, P. R. W. de Sauvage Nolting; *Poland*: I. Chlewicka, P. Brodzicki, T. Stasiuk, P. Szałkowski, W. Kulig, M. Maliszewski, K. Królicka, J. Zdrojewska, I. Nikodemska, A. Szpak, M. Wrębiak-Trznadel, A. Prokop, M. Szulc, A. Olszewski, W. Kępa, J. Banach, M. Węglarz, A. Gałuszka-Bilińska, A. Królak, E. Cisowska-Drozd, K. Orzechowski, M. Jeżewska, K. Adamaszek, G. Glanowska, T. Pitsch, G. Matuszewska, A. Nowowiejska-Wiewióra, M. Dereń, G. Walawski, M. Sołtysiak, R. Wysocki, G. Jarosiński, A. Drzewiecka, T. Ługowski, A. Jankowska, P. Błaszczak, J. Drozd, E. Łotocka, R. Duchowska, D.Sobczyk, P. Jarmużek, M. Sidor, D. Adamczyk-Kot, J. Sudnik, J. Cygler, I. Skoczylas, B. Poprawa, L. Kisiel, U. Kossowska, B. Sikorska-Buczkowska, K. Modzelewska, B. Demianiuk, W. Streb, T. Mularek-Kubzdela, P. Bogdański, E. Kaźmierczak, R. Zimoląg, J. Lorenc, R. Furtak, A. Regulska, M. Winter, M. Fic, P. Turek, E. Nowicka, W. Bryl, L. Lenartowska, O. Jerzykowska, M. Maćków, W. Gadziński, R. Kacorzyk, D. Zalewska, R. Sadłowski, J. Słaboszewska, M. Gruchała, A. Frankiewicz, J. Walczewska, A. Adamkiewicz-Piejko, R. Chyrek, L. Jankowska; *Portugal*: A. Correia, A. Girão, Á. Herdade, A. Sequeira, A. Tavares E Taveira, A. Gonzaga, A. Ribeiro, A. Albuquerque, A. Fernandes, A. Estriga, A. Rocha De Almeida, A. Lourenço, A. Pereira, A. Faria, B. Carvalho De Moura, C. Camossa, C. Alves, C. Aguiar, C. Rodrigues, E. Wellenkamp, E. Lins, F. Fernandes De Sousa, F. Moreira Pinto, F. Matias, G. Silva Alves, G. Bragança, G. Proença, G. Pêgo, H. Vinhas, I. Arroja, J. Rosa Pais, J. Morais, J. Silva E Sá, J. Vasconcelos, J. Matos, J. Freitas, J. Ferreira, J. Costa, J. Alcaravela, J. Mimoso, J. Antunes, J. Ferreira Dos Santos, J. Nobre Dos santos, J. Tito Martins, J. Fernandes, J. Chambel De Aguiar, J. Moreira, J. Carvalho, J. Forte De Carvalho, J. Calaça, L. Simões, L. Lopes Antunes, L. Soares, L. Semedo, L. Macedo, L. Sargento, L. Basto, L. Carpinteiro, L. Rebelo, L. Oliveira, M. Catarino Carvalho, M. Alves Costa, M. C. Gamboa, M. F. Ferrão E Vasconcelos, M. H. Custódio, M. I. Mendonça, M. J. Pinto Vaz, M. Espiga De Macedo, M. Lazaro, M. Martins Oliveira, N. Pelicano, N. Lousada, O. Rodrigues, P. Matos Dias, P. F. Fonseca, P. Ferreira, P. E. Abreu, P. Monteiro, R. Seabra Gomes, R. Carvalho, R. Santos, R. Pires Pereira, R. Rosado Soares, S. Baptista, S. Reis Monteiro, V. Gil, V. Sanfins, V. Martins; *Romania*: M. Anghel, C. Arsenescu Georgescu, K. Babes, M. Banu, R. Beyer, I. Bratu, A. Bumbu, R. Capalneanu, O. D. Chioncel, T. Chiscaneanu, R. Christodorescu, N. Cindea Nica, M. Cinteza, S. Coman, M. Constantinescu, E. Craiu, G. A. Dan, D. C. Dan, A. Dan, C. M. David, M. Dorobantu, D. Farcas, V. Firastrau, C. Florescu, A. Ghicu, A. Giuca, R. Grigoriu, D. A. Ionescu, D. D. Ionescu, L. C. Iosipescu, M. V. Ivan, D. Lighezan, S. Magheru, M. Magherusan, S. M. Marinescu, A. C. Motoc, R. Musetescu, M. Rau, L. Rotaru H. Rus, O. Sirbu, L. Sorodoc, C. M. Spinu, G. Stanciulescu, C. Statescu, M. Toringhibel, R. Trambitas, N. Trocan, A. Tudose, D. Vinereanu, M. Zagreanu,; *Russia*: D. Dymova, N. Semenova, A. Zherebtsova, V. Fedoskin, N. Gurianova, N. Bolotova, V. Knyazeva, T. Spitsina, N. Sytilina, N. Atamanchuk, M. Giorgadze, S. Zarechnova, S. Kutuzova, Y. Sharapova, I. Stelmakh, O. Sinyukova, S. Rostik, L. Evtukhova, L. Sukhanova, T. Makhieva, S. Tereshko, V. Kolesnikov, E. Kochurov, B. Marchenko, S. Nurgalieva, Z. Galeeva, E. Andreicheva, V. Zakirova, L. Baleeva, A. Minsafina, N. Borodina, Y. Arkhipova, T. Krechunova, M. Scherbak, A. Merkhi, N. Aksyutina, O. Ratovskaya, E. Suglobova, Y. Kozhelenko, E. Potapova, G. Poluyanova, N. Naberezhnova, E. Daniels, K. Atueva, L. Tsaryabina, A. Kurekhyan, N. Khishova, E. Dubinina, O. Demina, P. Mochkina, E. Bukanina, S. Tolpygina, Y. Polyanskaya, A. Malysheva, T. Kheliya, A. Serazhim, V. Voronina, Y. Lukina, R. Dubinskaya, N. Dmitrieva, M. Kuzyakina, N. Khartova, N. Bokuchava, E. Smirnova, A. Esenokova, Y. Pavlova, O. Smirnova, P. Astrakhantseva, S. Bykovskaya, O. Charikova, K. Berdnik, T. Karaseva, L. Zhabina, N. Oleinikova, O. Dzhkha, S. Grigoryan, E. Yakovenko, T. Ivaschenko, I. Kiseleva, T, Shokina, M. Novikova, A. Khodanov, L. Popova, L. Latyntseva, O. Kilaberiya, K. Makarenkova, N. Nosova, T. Gerasimova, L. Boikova, N. Sharapova, Y. Kulikova, , N. Pasechnaya, E. Bulakhova, S. Kurochkina, I. Bratishko, O. Likhobabina, E. Panova, N. Voronina, N. Bizyaeva, O. Gusev, N. Nevolina, T. Arsentieva, I. Budanova, E. London, Melnikova, A. Khripun, L. Polyaeva, E. Osadchuk, O. Krasnoslobodskaya, N. Yakimova, A. Lugin, Y. Sosnova, E. Il'ina, G. Kositsina, I. Shanina, S. Kostomarova, M. Malgina, M. Omelchenko, I. Gorlova, S. Eidelman, A. Salakhova, B. Bondarenko, R. Sopia, N. Baboshina, N. Eliseeva, F. Tumarov, N. Petrochenko, I. Khudina, N. Arabadzhi, V. Samakhovets, L. Tkhorzhevskaya, T. Sinotova, E. Zherlitsyna, S. Minkin, N. Petrova, Y. Tikhonov, N. Shmakova, V. Abduvalieva, M. Kuzmicheva, L. Nikolaeva, O. Varezhnikova, T. Dmitrieva, E. Mikhailova, Y. Yanina, L. Kapustina, Z. Vazhdaeva, G. Golovina, N. Fedorova, I. Nikolaeva, O. Fillipova, L. Gareeva, F. Tuktarova, N. Khmelevskikh, V. Karnot, M. Golub, I. Surovtseva, V. Kulygina, N. Shelomova, I. Kruglova, I. Pokrovskaya, O. Rodina, L. Polkina, N. Biryukova, E. Filippova, E. Kotova, T. Ignatieva, T. Alekseeva, L. Gruznykh, E. Mozerova, E. Moksyuta, E. Kosachek, N. Srtumilenko, O. Baranova, T. Voronova, L. Bayakhchan, I. Grudtsina, L. Gorshkova, O. Shamsutdinova, M. Getman, I. Gorodilova, N. Karnaukhova, V. Rotenberger, L. Isaeva, G. Lebischak, V. Ryzhkova, E. Usoltseva, D. Mescharekova, E. Tavlueva, E. Mineeva, M. Stikhurova, L. Kosareva, O. Grechishkina, S. Nikishina, A. Ilyukhina, O. Gureeva, I. Soin, S. Erofeev, S. Lebedev, l. Kudryavtsev, E. Gamzatov, N. Maximchuk, L. Grekhova, L. Kolevatova, M. Kazakovtseva, O. Kolesova, L. Zharikova, V. Kukaleva, N. Starostina, I. Grushetskaya, V. Kazachkova, I. Pashentseva, S. Shimonenko, I. Sirazov, A. Chernozemova, O. Golubeva, S. Mingalaeva, E. Zatsarina, D. Kozlov, N. Davydova, O. Larina; *Saudi Arabia*: K. Fayez Al-Habib, A. Al-Hersi, H. Al-Baker, H. Al-Faleh, A. Moberik, M. Radwan Arafah, M. Al-Shamiri, F. El-Shaer, M. Al Zaibag, M. Bdeir, I. Suliman, A. Mukhtar, H. Omar, A. Jamiel, A. Elkrail, M. Alanazy, M. Habab, K. Ashmak, R. Nourallah; *Singapore*: K. H. Mak, B. Singh, S. Baldev, T. S.Chee, C. C. Koo, L. P. Low, V. P. Nair, K. S. Ng, S. S. S. Quek, E. H. M. Tan, A. L. R. Ng, H. H. Chuang; *Slovakia:* G. Kamensky, G. Kaliska, J. Murin, K. Hatalova, L. Gaspar, I. Simkova, J. Dubrava, J. Pjontek, D. Pella, A. Banikova, M. Szentivanyi, F. Kovar, J. Benacka, I. Gonos, F. Fazekas, P. Kycina; *Slovenia*: J. Poles, Z. Fras, A. Pernat, A. Veternik, N. Černič-Šuligoj, M. Kerbev, I. Krajnc, P. Zagožen; *South Africa*: A. Alam, B Brown, B. Luke, E. Variava, R. Nethononda, S. Joubert, P. Matthews, L. Nkombua, V. Antia, D. P. Naidoo, J. Bhayat, S. K. George, N. Ranjith, G. H. M. Vawda, S. Govender, I. Soosiwala, K. Shein, M. Panajatovic, J. Flores, M. S. H. Khan, S. Blignaut, K. Coetzee, L. Burgess, V. Freeman, H. D. Theron; *Spain*: M. A. Arnau Vives, F. J. Abardía Oliva, V. Albero Martínez, J. M. Alegret Colomer, E. Alegría Ezquerra, C. A. Almeida Fernández, N. Alvarenga Recalde, A. Alvarez Auñon, P. Alvarez García, C. Amo Fernández, C. Amoros Galito, R. Ancín Viguiristi, J. Antona Makoshi, M. Aparici Feal, A. Ardiaca Capell, J. Arnedillo Pardo, G. Arquero García, V. Arrarte Esteban, M. Baquero Alonso, P. Barahona Pérez, J. L. Bardají Mayor, V. Barriales Alvarez, A. Batalla Celorio, D.Bierge Valero, J. Blanco Castiñeiras, F. Bosa Ojeda, C. Botana Penas, H. Brufau Redondo, J. Bruguera Cortada, J. Cabau Rubies, R. Cabrera Solé, F. Calvo Iglesias, S. Cantabrana Miguel, R. Carrillo Cardoso, M. Casanovas Pié, P. Casas Giménez, E. Castillo Lueña, J. A. Castillo Moreno, M. Castillo Orive, A. Chirivella González, J. M. Chopo Alcubilla, V. Climent Payá , M. A. Cobos Gil, J. L. Colomer Martín, A. Concepción Clemente, R. Cortés Sánchez, D. Cremer Luengo, S. Darnes Soler, J. de Andrés Novales, R. De Castro Aritmendiz, J. L. Delgado Prieto, J. L. Díaz Díaz, C. Escobar Cervantes, J. Ezcurdia Sasieta, L. Facila Rubio, C. Falces Salvador, P. Federico Zaragoza, R. Fernández Alvarez, F. Fernández de la Cigoña, L. A. Fernández Lázaro, L. C. Fernández Léoz, R. Fernández Mouzo, M. Fernández-Valls Gómez, B. Ferreiro Rodríguez, C. Franco Aranda, J. Freire Corzo,J. Fuertes Alonso, J. Fuertes Beneitez, E. Galve Basilio, C. García García, M. J. García González, S. García Ortego, V. García Saavedra, J. García-Moll Marimón, R. Gascueña Rubia, D. Gentille Lorente, H Gervas Pavón, R. Gilabert Gómez, J. J. Gómez Barrado, J. J. Gómez Doblas, M. J. Gómez Martinez, C. González Juanatey, V. González Toda, M. Gonzalvez Ortega, E. Gordillo Higuero, J. Hernández Afonso, D. Herrera Fernández, E. Homs Espinach, A. Idoate Gastearena, M. Irurita Latasa, R. Izquierdo González, M. Jaquet Herter, M. Lagares Carballo, J. A. Lastra Galán, B. Limeres González, M. A. López Aranda, L. López Barreiro, D. López Gómez, A. López Granados, V. López Mouriño, J. L. López-Sendón, L. Mainar Latorre, E. Marín Araez, F. Marín Ortuño, A. Martín Santana, J. Martínez Florez, J. Martínez González,J. F. Martínez Rivero, D. Marzal Martín, G. F. Mazzanti Mignaqui, A. Melero Pita, E. Molina Laborda, Mª A. Montero Gaspar, J. Mora Robles, J. Morales González, J. Moreno Arribas, Mª T. Moreno Casquete, J. A. Moro López, C. Moya López, N. Murga Eizagaechevarría, F. Narro García, J. Navarro Manchón, C. Navas Navas, E. Novo García, J. A. Núñez Gamero, A. Ordóñez España, J. A. Ortiz de Murua López, E. Orts Soler, E. Otero Chulian, L. Pastor Torres, A. J. Paule Sánchez, M. A. Paz Bermejo, G. Peña Pérez, J. Á. Perea Egido, L. Pérez de Isla, S. Pérez Ibiricu, Mª A. Pérez Martínez, M. Pérez Paredes, E. Peris Domingo, J. Pinar Sopena, C. Pindado Rodríguez, Mª J. Pinilla Lozano, C. Piñero Ramírez, Y. Porras Ramos, F. Ramos Ariznabarreta, M. Rayo Gutiérrez, J. M. Roca Catalán, A. Rodríguez Almodóvar, J. Rodríguez Collado, A. Rodríguez Fernández, J. A. Rodríguez Fernández, J. A. Rodríguez Hernández, I. Rodríguez Tejero, I. Romeo Castillejo, D. Romero Alvira, J. A. Romero Hinojosa, C. Romero Menor, P. Rossi Sevillano, E. C. Rueda Calle, J. Rueda Soriano, P. Ruiz Pérez, T. Sagastagoitia Gorostiza, I. Sainz Hidalgo, M. Sandin Rollán, S. Santaolalla Rodríguez, E. Santas Olmeda, J. L. Santos Iglesias, M. L. Sanz Rodríguez, I. Segura Laborda, S. Serrano García, B. Sevilla Toral, L. Silva Melchor, E. Simarro Martín-Ambrioso, R. Sola Casado, C. Soriano Navarro, Mª I. Soto Ruiz, P. Talavera Calle, P L. Torres Díaz, A. Troncoso Gil, F. Trujillo Berraquero, M. A. Ulecia Martínez, J. Umaran Sánchez, C. Vaticón Herreros, A. Vázquez García, J. L. Vega Barbado, E. Velasco Espejo-Saavedra, T. Vicente Vera, M. Vida Gutiérrez, C. Villar Mariscal, G. Vives Bonato, L. Wu Amen, G. Yanes Bowden, J. C, Yañez Wonenburger, J. L. Zamorano Gómez, J. Zarauza Navarro; *Switzerland*: P. Monnier, A. Jaussi, A. Forclaz, M. Grobéty, L. Schlueter, C. Vuille, C. A. Nacht, D. Evéquoz, S. Ciaroni, F. Dominé, J. Bérubé, H. Rickli, J. Hellermann, R. Koller, G. Bourgeois, R. Engel, C. Niederberger, P. Stadler, M. Gnädinger, C. Schmied, T. Wettstein, C. Badorff, P. Hilti, C. A. Chételat, F. Sepulcri, H. Brunner, J. Schindler, M. Kraus, W. Gmür; *Thailand*: C. Bouranasompop, W. Jiraroj-ungkun, W. Lapanun, V. Vivekaphirat, S. Panpunnung, S. Dutsadeevettakul, S. Tasneeyapant, P. Ngamjanyaporn, S. Apitamsuntorn, W. Tantisiriwat, T. Suithichaiyakul, S. Kuanprasert, W. Wongcharoen, A. Phrommintikul, C. Musigchai, T. Chantrarat, P. Uerojanaungkul, S. Apinyasawat, T. Tangcharoen, M. Lertnantakul, A. Wasuwat, J. Harinasuta, O. See, V. Chaithiraphan, T. Boonyasirinant, W. Boonyapisit, M. Kittipovanonth, A. Buakhamsri, D. Piyayotai, P. Hutayanon; *UK*: S. Junejo, O. Aiyegbayo, H. Ancliff, C. Bradshaw, R. Cervenak, H. Choi, E. George, I. Gilmour, D. Gough, A. Idrissi-Sbai, J. Ingham, B. Al-Khalidi, A. Liston, J. Mackrell, I. Pattison, R. Ramachandran, N. Ray, G. Reddy, I. Sen, K. Shetty, L. Singh, M. Stanley, A. Wallace, M. Weatherhead, T. Gilbert, G. McCansh, S. Higgins, C. Killeen, I. Cromarty, P. Franklin, E. Pinch, A. Dhesi, C. Dernedde, M. Lawrence, H. Simper, M. Noble, G. Dalton, L. Stevens, P. Berry, C. Hand, R. Oliver, H. Jones, P. Sampson, N. Taylor, R. Grogono, J. Dalrymple, A. Martin, S. Thurston, K. Elsby, M. Vallis, G. Morrison, C. Lang, A. Watson, A. Thomson, H. Dougall, B. La Hay, L. Compson, A. McCracken, J. Calder, F. Weber, D. Richmond, R. Brownlie, G. Brown, H. MacCowan, A. Heap, M. Perry, L. A. Holden, G. Scott, N. Haldane, S. Hood, I. Cullen, J. Bell P. McNaught, M. Sharif, J. Dunn, D. Hay, S. Ross, R. Shaw, L. Hay, S. Langridge, R. Burns, L. Crawford, A. Kennedy, D. Logan, P. McAlavey, M. Brown, P. Costello, G. McLaren, A. Potter, J. McPherson, M. Drijfhout, J. Finlayson, D. Troup, A. Woodall, J. Pearce, S. Williams, W. Parkar, A. Yusuf, I. Benett, P. Bishop, H. Thomas, I. Caldwell, P. Ormiston, S. Kwok, S. Wright, N. Kanumilli, P. Saul, H. Milligan, I. Wilkinson, A. Vance, N. Paul, C. Paul, I. Shaikh, R. Ellis, N. Vites, R. Steeds, D. Goodwin, A. Aftab, S. Banham, N. Chauhan, M. S. Grocutt, A. Gupte, R. Jordan, B. S. Jheeta, K. Ladha, M. Nazir, R. Pal, R. P. Patel, R. McManus, A. Singal, P. Saunders, A. B. Syed, A. Bahal, H. Dau, D. M. Walker, R. McNeilly, A. Bolidai, N. MacCarthy, D. Lawton, M. Vardhani, G. Sengupta, D. Kinloch, F. Howie, A. Serrano-Garcia, S. E. Paget, R. Till, P. Seal, J. Morrell, T. Maxwell, G. Singh, D. Warden, R. Elias, C. Dixon, R. K. Pandey, V. Challenor, S. Davies, M. Gibbs, A. Gillet, C. Goldie, I. Jarvis, P. Johnson, M. Malden, J. Moore, C. Morton, K. Nehrig, P. Sheringham, G. Wilson, J. Halcox, I. O'Connor, K. Ling, D. Edwards, H. Charles, A. Weatherup, E. Davies, N. Watkins, D. Morgan, R. Davies, A. Lindsay, D. Beacock, R. Balai, P. Kirmond, P. Brindle, C. Bundy, T. Cahill, A. Dayani, P. Eavis, S. Mohr, S. Hayne, C. Krasucki, M. Micheals, I. Orpen, I. Parker, R. Sewell, D. Sharp, A. Smith, A. Stevens, J. Upton, J. Victory, C Wernham, R. Davis, C. Mays, M. Andrews, J. Takhar, C. Travill, P. Choudhury, W. Matta, A. Ihonor, C. O'Dong, S. Rahman, P. Singer, S. Gillam, P. S. Bath, N. Razzaq, O. O'Toole, P. Rowe, H. Williams, P. Kalra, A. Allcock, A. Tucker, V. Sprott, K. Kyd, G. Cunliffe, C. Arden, A. Bateman, G. Kassianos, D. Sinclair, C. Turner, R. Jagathesan, F. Sattar, A. Ashford, A. Chukwu, H. Taylor, R. Pradhan, T. Rundell, R. Howlett, R. Bietzk, R. Patel, M. Myint, M. Partington, F. O'Reilly, M. Baverstock, S. Dixon, M. Tennekoon, N. Brand, P. Haimes, P. Keller, S. Whetstone, R. Davis, C. Mays, M. Andrews, J. Takhar; *Ukraine*: O. Kovyrshyna, V. Rogozhyna, T. Kiver, V. Vasylenko, L. Kucheryava, S. Salimova, V. Alekseenko, O. Gukov, I. Myhailiv, L. Kardashevskaya, O. Prikolota, O. Bashkirtcev, E. Andreev, L. Tkachenko, M. Mospan, V. Batushkin, L. Safonova, A. Ogorodnichuk, S. Pustovit, S. Romanov, L. Burlakova, Y. Voloshko, V. Lafarenko, Z. Vlasuk, O. Leshchuk, S. Chushak, V. Koval, O. Stasuk, O. Pogrebna, S. Kornienko, S. Tikhonova, T. Fesenko, T. Kuzmina, O. Ushakov, N. Vechtomova, L. Potapska, I. Illushechkin, E. Kryvenkova, O. Lysunets, O. Tsygankov, L. Bardachenko, L. Voloshyna, V. Ginzburg, L. Franskyavichene, T. Korotich, N. Vyshnevaya, N. Bilous, S. Kulinich, V. Kulik, I. Sadykova, T. Berezhna, S. Molotyagina; *Vietnam*: L. V. Nguyen, M. H. Pham, H. T. Pham, N. H. Khong, K. B. Do, T. B. LE, P. A. Do, T. C. Do, N. Q. Nguyen, Q. H. Do, K. C. Vu, N. H. Pham, T. H. T. Pham, M. C. Ta, D. P. Phan, T. T. H. Nguyen, T. T. N. Pham, T. L. To, V. T. Le, L. Dang, L. Bui, T. T. H. Pham, H. H. Phan, T. T. H. Bui, T. V. A. Tuong, T. P. Nguyen, T. H. Nguyen, B. K. Nguyen, D. B. Vu, N. S. Pham, T. Q. Do, T. S. Pham, V. D. Dang, D. T. Le, V. C. Do, T. K. L. Nguyen, H. D. Luong, T. Q. Luu, N. V. Pham, T. K. Huynh, N. T. H. Tu, K. A. Ngo, T. T. C. Nguyen, T. T. L. ONG, V. B. Doan, T. B. Kim, T. N. Vo, T. T. T. Tran, T. A. Nguyen, V. D. Tran, A. K. Nguyen, A. C. Tran, M. H. Ngo, N. H. Vu, I. T. Ly, N. P. H. Tran, L. U. P. Tran, T. N. Nguyen, T. H. Tran, P. H. Truong, T. L. Mai, V. S. Hoang, C. M. A. Bui, V. P. Dang, Q. B. Truong, M. P. Vo, V. T. Nguyen, N. H. Chau, T. T. H. Ta, H. N. Dinh, H. Tran, H. K. N. Nguyen; *West Indies*: A. Chung, E. Chung, B.Martina-Hooi, R. Angela, P. Ramoutar, R. Fillet, R. Tilluckdharry, T. Dookie, E. Foster, C. Hart, F. Omardeen, S. Ramphall, C. Lalla, R. Henry, J. Cheng, V. Elliott, H. Falconer, L. Hurlock-Clarke, R. Ishmael, G. Lalljie, K. Lee, A. Liqui-Lung, R. Massay, H. Mohammed, C. Brown, R. Daniel, M. Didier, Z. Salas.
